# Physical activity promotion and health-enhancing physical activity education policy in EU healthcare: a cross-sectional survey of 27 member states

**DOI:** 10.1136/bmjopen-2024-095218

**Published:** 2025-08-13

**Authors:** Callum Leese, Stephen Whiting, Romeu Mendes

**Affiliations:** 1 School of Medicine, University of Dundee College of Medicine Dentistry and Nursing, Dundee, UK; 2 Division of Noncommunicable Diseases and Promoting Health through the Life-course, World Health Organization Regional Office for Europe, Copenhagen, Denmark; 3 World Health Organization Regional Office for Europe, Copenhagen, Denmark; 4 Universidade do Porto, Porto, Portugal; 5 Unidade Local de Saúde de Trás-os-Montes e Alto Douro, Vila Real, Portugal

**Keywords:** Health policy, PUBLIC HEALTH, Exercise

## Abstract

**Abstract:**

**Objectives:**

Analyse data collected through the WHO Regional Office for Europe to describe the proportion of European Union (EU) member states that have relevant policies related to physical activity (PA) counselling and exercise referral schemes in healthcare settings and the education of health professionals in health-enhancing PA (HEPA).

**Design:**

Cross-sectional survey.

**Setting and participants:**

An online survey (LimeSurvey) was sent to nominated government representatives of the 27 EU member states (via the EU PA Focal Point Network) in March 2021. The survey was open for 2 months, with support offered to all national representatives by the WHO Regional Office for Europe throughout. The survey had been developed by a panel of experts and was previously disseminated (and analysed) in 2015 and 2018.

**Outcomes measures:**

National recommendations regarding (1) PA counselling in healthcare settings and (2) the inclusion of HEPA within teaching curricula.

**Results:**

All 27 countries responded. Of the 18 countries that reported national policies to provide PA counselling by healthcare professionals (HCPs), all reported that counselling on PA was provided through primary care, with an additional half also reporting PA counselling provision as part of secondary care. Twenty-one countries reported that HEPA is taught in the curricula of HCPs, but large variations exist regarding which cadres of HCPs have HEPA integrated within their curricula and whether the HEPA teaching is a mandatory or optional component of training.

**Conclusions:**

Despite PA counselling being a key recommendation for promoting PA at the population level, only two-thirds of EU member states have national policies in place. Although three-quarters of EU member states report healthcare education curricula, including HEPA, more research is required to understand the methods and content of delivery and the subsequent effectiveness on knowledge and clinical outcomes.

**STRENGTHS AND LIMITATIONS OF THIS STUDY**

- This study is unique in presenting data on the prevalence of physical activity (PA) promotion and training in the curricula of health professionals among each of the European Union member states.
- All responses are based on a single expert response from national governments, who may have a limited overview.
- Study data are limited to qualitative responses from surveys, with no direct information available on what the countries actually do in practice.
- The data collection on health-enhancing PA indicators in March 2021 may have been affected by the COVID-19 pandemic.

## Introduction

 Physical activity (PA) promotion delivered via primary care has been shown to be effective at initiating behavioural change and reducing the risk of disease progression.^1 2^ In most countries, primary care professionals are the first point of contact individuals have with the health system, providing greater exposure to the whole population than any other health professionals.^3 4^ They also regularly see those in need of PA advice and are viewed by the public as a trusted source of information.^3 4^ PA promotion in healthcare settings can take a number of different formats, but in general refers to PA counselling, PA on prescription or exercise referral.^5^ PA counselling involves using all or part of a patient consultation to change PA behaviour for the prevention (primary or secondary) of associated chronic health conditions.^6^ PA on prescription involves healthcare professionals (HCPs) providing individually tailored prescriptions for PA, but is preceded by a person-centred dialogue and involves structured follow-up.^7^ Finally, although the nature of exercise referral schemes varies, it involves individuals with disease or disease risk factors (who would benefit from increased PA) being referred by HCPs to a service that delivers and monitors an exercise programme tailored to that individual's needs.^8^

Research has shown PA promotion interventions within primary care to be cost-effective,^9^ with a shifting emphasis within healthcare settings from treatment to prevention.^10^ Acknowledging this, WHO Europe highlighted PA counselling in primary care as one of its ‘best buys’ in an economic analysis of cost per disability-adjusted life year averted.^11^

Despite this, implementation of PA promotion via primary care is limited,^12^ with a lack of support and education of health professionals often cited as an explanation.^13^,^17^

Insufficient PA is now a well-known public health issue, and governments and organisations are increasingly creating policies and programmes to address it.^18^,^20^ Acknowledging the value of a European Union (EU) wide collaborative approach, in 2008, the European Commission published *EU Physical Activity Guidelines: Recommended Policy Actions in Support of Health-Enhancing Physical Activity*^19^ in order to support implementation of PA policies at the national level. In 2013, EU Member States adopted the Council of the EU recommendation on *promoting health-enhancing physical activity across sectors*^21^ and the health-enhancing PA (HEPA) Monitoring Framework to monitor implementation of the EU PA Guidelines,^22^ with the European Commission and the WHO Regional Office for Europe subsequently supporting a collaborative survey-based project to monitor HEPA policies in the EU Member States.

Since 2015, in 3-yearly cycles, the European Commission, in collaboration with the WHO Regional Office for Europe, has been monitoring the implementation of HEPA policies in EU member states.^23^,^25^ The published reports offer a limited overview of HEPA policies. Of relevance, they report 75% (n=21) of countries had a national programme or scheme for the provision of counselling on PA by health professionals in 2021, compared with 67% (n=18) in 2018. Similarly, in 2021, PA for health was included in the curricula of one or more types of health professionals in 78% (n=21) of the included countries, compared with 61% (n=17) in 2015 and 79% (n=22) in 2018.

Given the previously limited data availability on HEPA policies in healthcare settings, this report aims to analyse data collected through the HEPA Monitoring Framework to describe the proportion of EU member states that have relevant policies related to PA counselling and exercise referral schemes in healthcare settings and the education of health professionals in HEPA.

## Methods

As previously described, the implementation of HEPA policies in EU member states has been monitored in 2015, 2018 and 2021.^23^,^25^ More information regarding definitions used, operationalisation and data sources on indicators can be found in the European Commission Staff Working Document on the HEPA Monitoring Framework.^22^ Twenty-three indicators of the HEPA Monitoring Framework exist, covering national recommendations on PA, PA surveillance, levels of engagement in PA, coordination and funding, awareness raising and policy implementation in different settings, such as sport, health, school, workplace, urban planning and special populations. Of the 23 indicators, across 10 thematic areas, indicators 10–12 are related to promoting PA through the health sector.

For the 2021 round of data collection, an electronic, English-language questionnaire was developed by experts within the WHO Office for the European Region in January 2021, using LimeSurvey software. It was distributed to the 27 EU member states in the WHO European Region through the EU PA Country Focal Points Network at the beginning of March 2021. This is a network of experts officially nominated by their governments to support data collection on HEPA promotion at the national level. These experts usually work in national ministries of health, ministries of sport or related national agencies. The cross-sectional survey was open for 2 months, and during this time, the WHO maintained a helpdesk and held three webinars to provide guidance to officials nominated as part of the Focal Point Network. On closing, all 27 countries had responded. WHO staff then manually checked all responses and contacted corresponding focal points if further clarification was needed. Although summary findings were reported in the 2021 report on HEPA in the EU,^24^ this study further explores the responses to survey questions regarding policy implementation in healthcare settings (indicators 11 and 12).

Specifically, the questionnaire (detailed in online supplemental file 2) asked about the existence of national guidance or programmes for PA counselling by HCPs, the involvement of various health professionals, financial incentives and whether patients incur costs for such services. Additionally, the survey covered the integration of HEPA in the curricula of health professionals, specifying professional groups, study levels and whether such education is mandatory or optional. Respondents were also invited to share relevant success stories or case studies. Country focal points answered the questionnaire based on the available data in that country. Quantitative and qualitative data from the questionnaires were descriptively analysed and compared.

### Patient and public involvement

None.

## Results

### National guidance or programme to promote counselling on PA and/or an exercise prescription

18 of the 27 countries reported having national guidance or a programme to promote counselling on PA and/or exercise prescription by HCPs (table 1). All reported national guidance or programmes were based in primary care, with 9 (50%) also including secondary care settings.

**Table 1:** Summary of the national PA counselling or exercise prescription programmes reported by EU member states

Country	National system	Service delivery location	HCP financial incentive	Patient financial contribution
Austria	N	N/A	N/A	N/A
Belgium	Y	P	Y	Y (Reim)
Bulgaria	Y	P	N	N
Croatia	Y	P	Y	N
Cyprus	N	N/A	N/A	N/A
Czechia	N	N/A	N/A	N/A
Denmark	Y	P/S	N	N
Estonia	*N*	N/A	N/A	N/A
Finland	Y	P/S	N	N
France	Y	P/S	N	N
Germany	Y	P/S	N	N
Greece	N	N/A	N/A	N/A
Hungary	Y	P	N	N
Ireland	Y	P/S	N	Y (Reim)
Italy	N	N/A	N/A	N/A
Latvia	Y	P	N	N
Lithuania	Y	P	N	N
Luxembourg	N	N/A	N/A	N/A
Malta	N	N/A	N/A	N/A
The Netherlands	Y	P/S	N	N
Poland	Y	P/S	N	N
Portugal	Y	P	N	N
Romania	N	N/A	N/A	N/A
Slovakia	Y	P	N	N
Slovenia	Y	P	Y	N
Spain	Y	P/S	N	N
Sweden	Y	P/S	N	N

EU, European Union; HCP, healthcare professional; N, No; N/A, not available; P, primary care; PA, physical activity; P/S, primary and secondary care; Reim, re-imbursed; S, secondary care; Y, Yes.

Three (17%) of the 18 countries with national guidance or programmes reported financially incentivising HCPs for providing PA counselling or exercise prescriptions. Two countries reported charging patients for counselling or exercise prescriptions, although in both countries, the money can be reclaimed through insurance.

### PA and health education in health professional curricula

Twenty-one (78%) of the EU Member States reported that PA and health are taught in the curricula of HCPs.

**Figure 1:**
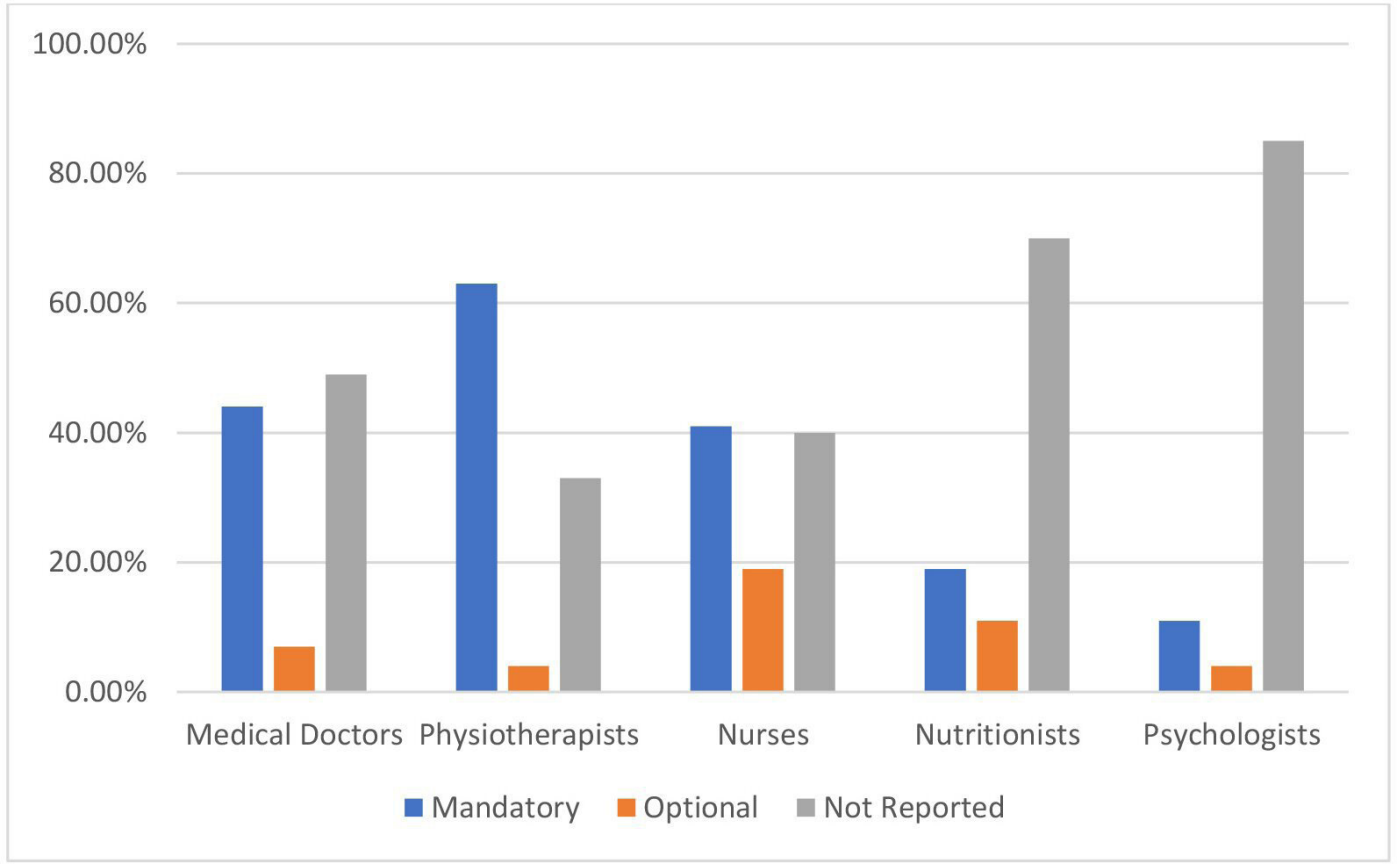
Percentage of EU countries (n=27) that report delivering physical activity in the curricula of health professionals, separated by health-professional groups and whether it is mandatory or optional for undergraduate curricula.

**Figure 2:**
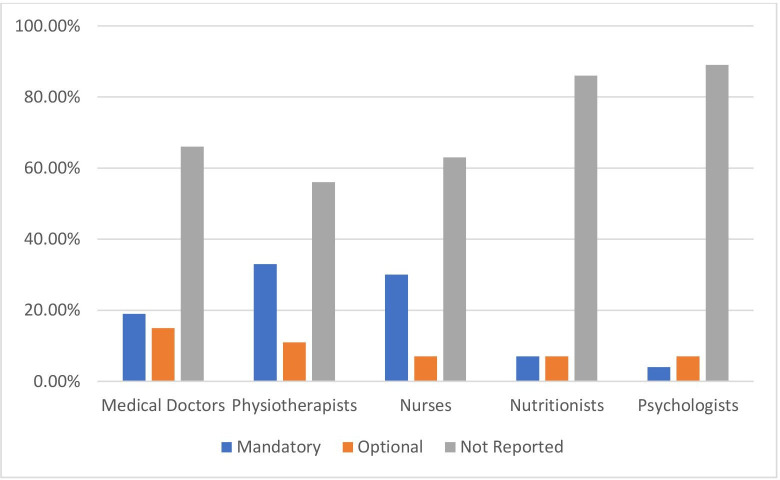
Percentage of EU countries (n=27) that report delivering physical activity in the curriculum of health professionals, separated by health professional groups and whether it is mandatory or optional for postgraduate curricula.

Figure 1 shows the percentage of EU countries (n=27) that report delivering PA in the curricula of health professionals, separated by health professional groups and whether it is mandatory or optional for undergraduate curricula. Figure 2 represents the same data but for postgraduate curricula.

As seen in figure 1, large variation exists in whether PA is taught in undergraduate curricula between health professionals and whether it is mandatory or optional. Eighteen (67%) countries reported PA education (both mandatory and optional) in the undergraduate curriculum of physiotherapists, compared with 60% (n=16) for nursing students and 51% (n=14) for medical students.

Similar patterns existed in postgraduate curricula (see figure 2). Twelve (44%) countries reported PA education (mandatory and optional) in the postgraduate curriculum of physiotherapists, with 37% (n=10) for postgraduate nursing students and 34% (n=9) for medical students.

Countries also reported on other professions, including nutritionists (30% of countries at the undergraduate level and 14% at the postgraduate level) and psychologists (16% of countries at the undergraduate level and 11% at the postgraduate level).

Online supplemental file 1 includes a number of case studies, highlighting positive examples from across EU member states.

## Discussion

The study summarises the current monitoring of HEPA indicators for PA promotion in healthcare settings. With all EU member states’ data included within the study, it gives a comprehensive overview of the relevant HEPA indicators (11 and 12): counselling on PA and training on PA in curricula for health professionals.

This research shows that, despite counselling on PA in primary care being a key recommendation for population-based PA promotion,^11^ only two-thirds of EU member states have national policies in place, with integration of education in PA for health into HCPs’ curricula being limited. Building on previous work,^26^ a recent publication by Whiting and colleagues^27^ has highlighted the ongoing gap between PA policy and PA implementation. There are a number of challenges, including a lack of evidence to inform PA policy,^28^ poor translation of effective research into practice (including accounting for country-specific and culture-specific factors) and a failure to implement effective strategies.^27^ This may be further complicated by a lag time between implementation and results and a large number of confounding factors, including economic developments, social trends, climate change and more recently the COVID-19 pandemic.^27^

### Counselling on PA

In 2018, the WHO launched the ‘Global Action Plan on PA 2018–2030’,^29^ with action point 3.2 calling for the implementation and strengthening of ‘*systems of patient assessment and counselling on increasing physical activity… appropriately trained health, community and social care providers*’. This action point is in response to growing evidence supporting the effectiveness of PA promotion delivered in primary care at increasing PA in patients, with a relative risk, compared with no advice, of 1.22–1.42.^1 5 30 31^ The WHO highlighted PA promotion as a ‘best-buy’ through a cost-effectiveness analysis.^11^

Despite this, PA promotion in primary care remains poorly implemented. For example, 72% of general practitioners (GPs) in England self-report that they do not deliver PA promotion.^12^ A recent systematic review explored factors associated with the delivery and receipt of PA advice and support in primary care.^15^ Key practitioner barriers included a lack of time and training/guidelines and a perceived lack of patient motivation/adherence to PA advice. Additionally, organisational factors included economic constraints and a lack of support from management. The challenges are consistent across a number of other reviews.^32 33^ These findings, and particularly the organisational challenges, highlight the need for policy implementation and support. The fact that only two-thirds of EU member states have national guidance or a policy in place for counselling on PA and/or an exercise referral scheme highlights that there is room for ongoing improvement. All national programmes or policies reported in this study involved primary care settings, with nine also including secondary care. Although the vast majority of the research has been performed in primary care, a recent^34^ report identified the need for integration of PA promotion across primary and secondary care. This is particularly relevant in long-term conditions (eg, cardiovascular disease and diabetes) where care is shared between both primary and secondary care, and the continuity of messaging is of greater importance. In discussing the PA advice continuum, Freene and colleagues^35^ highlight the importance of PA promotion by the full spectrum of care providers, regardless of discipline or availability of time. In the development of the ‘Active Hospitals’ toolkit,^36^ Moving Medicine and the Faculty of Sport and Exercise Medicine are actively trying to address this in the UK, complementing pre-existing initiatives in primary care.^37 38^ More research is required to explore the role of secondary care in PA promotion and its integration with other healthcare settings.

In this report, only three countries reported using financial incentives as part of a national programme to encourage PA promotion. In 2022, the WHO published a manual^10^ to guide the implementation of ‘brief’ behaviour change interventions in primary care, including PA. To embed these interventions, it identified six building blocks for structural support, including financial incentives for primary care-based programmes. Financial incentivisation has previously been used effectively in the provision of smoking cessation through primary care,^39^ and given that brief advice for PA is reportedly comparable at inducing behaviour change to brief advice for smoking cessation, the benefits of incentives through primary care could be significant.^5 40^

### Training on PA in the curricula for health professionals

The 2018 Global Action Plan on PA^29^ also calls for the training of professionals within the health sector to increase knowledge and skills(in Action 1.4). The failure of medical education to address PA for health has been repeatedly highlighted since 2000,^41^,^45^ with an editorial published in 2023 calling for the issue to be readdressed in response to a lack of change.^46^ Despite the mounting evidence and calls from international organisations to address the lack of education in healthcare curricula, this study highlights a lack of routine inclusion within undergraduate and postgraduate curricula across all health specialities. This lack of standardisation was highlighted by Netherway and colleagues,^47^ who called for the development of guidelines and best practices. The VANGUARD project^48^ is an ERASMUS+ Sport collaboration between five EU countries, seeking to address this by creating and implementing resources (#MovementforMovement) and educating future health professionals about PA for health.^49^ All educational institutions for health professionals within EU member states need to accredit programmes in accordance with the EU guideline 2005/36/EG,^50^ but within this, there are no specific indications for HEPA and its education. Given the need to address physical inactivity and existing evidence highlighting a lack of education on PA for health as a major barrier to delivering PA promotion, there is potential to update the EU legislative guidelines for the accreditation of healthcare programmes, seeking the inclusion of education in HEPA. To improve the mandate for this further research, we could look to create a robust economic argument for the inclusion of teaching within healthcare curriculums, addressing a current evidence gap.

### Limitations

This study is, to the best of our knowledge, unique in presenting data on the prevalence of PA counselling and training on physical activity in curricula for health professionals among EU member states. There are, however, a number of limitations. First, all responses are based on a single expert response from national governments, who may have a limited overview. The quality and depth of these responses are inherently dependent on the knowledge of the focal points within the EU PA Focal Point Network. Given the variation within educational institutions within a single country, the responses are also likely influenced by the access to detailed curricular information from relevant professional training institutions that the local focal point responder had available. Therefore, while we report the proportion of countries indicating inclusion of HEPA, we acknowledge that the scope likely ranges from general education on PA and its health impacts to more applied or clinical content, such as counselling techniques or prescription practices.

Furthermore, study data are limited to qualitative responses from surveys with no direct information available on what countries actually do. Challenges in the interpretation of responses exist, with responses of ‘no’ or ‘-’ either being an affirmative response or an indication that the responding expert is not sure if policy exists. In addition, the study method may have limited response depth. For example, the survey did not provide a detailed breakdown of specific educational content areas, such as whether curricula included training on behaviour change techniques, patient counselling or intervention delivery.

Finally, the data collection on HEPA indicators in March 2021 may have been affected by the COVID-19 pandemic, with the changing face of healthcare delivery and healthcare education in light of the pandemic potentially impacting national policy.

### Implications for future research and policy

This study highlights a number of opportunities for future research. First, a deeper understanding of the national programmes and policies being implemented, and their subsequent effectiveness, would allow for a deeper understanding of what is working within the context of a systems-based approach. A first step to address this would be to conduct a more thorough evaluation of individual countries’ policies, allowing for triangulation with these results. Furthermore, research is required to explore the role of secondary care (and its integration with primary care) in PA promotion, as well as the impact of financially incentivising health professionals for the promotion of PA. Finally, further research is required to explore how HEPA is taught across healthcare curricula and the subsequent impact on knowledge of HEPA within HCPs and clinical outcomes in patients.

Netherway and colleagues^47^ also outline that established healthcare curricula are at capacity, with little room for additional material. National policy to mandate training to HCPs in training, like the Irish ‘Making Every Contact Count’ training programme,^51^ might ensure implementation, but thought must be taken to consider the cost. At an EU level, changes to the aforementioned guideline 2005/36/EG^50^ would have a similar effect.

## Conclusions

This study highlights that two-thirds of EU member states have implemented national guidance or programmes promoting PA counselling and exercise prescription. More work is needed to explore the role of secondary care in PA promotion and the effectiveness of incentivising HCPs. Additionally, only 78% of countries include PA education in healthcare curricula, with considerable variability between professions and educational levels. Future research should focus on evaluating the impact of education on HCPs’ competence and patient outcomes.

## Supplementary material

10.1136/bmjopen-2024-095218 online supplemental file 1

10.1136/bmjopen-2024-095218 online supplemental file 2

## Data Availability

Data are available upon reasonable request.
