# Supplementary material for: Physical activity promotion and health-enhancing physical activity education policy in EU healthcare: a cross-sectional survey of 27 member states
Source: BMJ Open. 2025 Aug 13;15(8):e095218. doi: 10.1136/bmjopen-2024-095218 (PMC12352183; doi:10.1136/bmjopen-2024-095218)
Supplement: online supplemental file 1 [file bmjopen-15-8-s001.docx]

Supplementary File: Case Studies

*Highlight 1*

Of the 18 countries that reported national guidance or programmes, 3 countries reported legislative changes to legally protect counselling on physical activity or exercise prescription in healthcare settings. These countries were: (1) Germany, which introduced the *Act to Strengthen Health Promotion and Preventive Health Care (Preventive Health Care Act)* in 2015, (2) Bulgaria with the legislative *Ordinance Number 8 for Prophylactic Medical Examinations and Screening* in 2016 and (3) France, with a national decree on “*Physical Activity Prescription*” in 2016.

*Highlight 2*

Two countries have been highlighted due to the national programme they have reported to have implemented for the education of health-professionals:

1. Austria, reported the highest number of mandatory hours of teaching of health-professionals from all EU Member states included within the report, with 20 hours of mandatory teaching to medical students in PA and health.
2. Romania, reported a government published curriculum mandate for medical schools to cover, including physical fitness and PA learning outcomes (25). This was the only nation that reported doing so in the survey.

*Highlight 3*

With most countries highlighting the autonomy of the individual or educational institution to pursue option PA and health education, the plethora of options available can present challenges. To address this Czechia was the only country that reported to have implemented an authorisation for courses through the ministry for health (26). This in theory provides a level of standardisation and validation, benefit patient and practitioner alike.
