# Supplementary material for: Physical activity promotion and health-enhancing physical activity education policy in EU healthcare: a cross-sectional survey of 27 member states
Source: BMJ Open. 2025 Aug 13;15(8):e095218. doi: 10.1136/bmjopen-2024-095218 (PMC12352183; doi:10.1136/bmjopen-2024-095218)
Supplement: online supplemental file 2 [file bmjopen-15-8-s002.docx]

***Supplementary File 2: ‘Physical Activity Data Collection Tool’ Questionnaire.*  Questions for Indicator 11 and 12: Counselling on physical activity and exercise prescription**

9.1 Is there any national guidance or programme to promote counselling on physical activity or exercise prescription by health professionals in your country? Y/N

9.1.a Can you please comment on why you have answered No?

9.1.b If Yes, please provide a short description, including which professionals are involved, if implemented in the public and/or private sector, and in primary health care and/or hospitals.

9.1.c Are there any financial incentives provided to health professionals to encourage patients to be more active? Y/N

9.1.c.1 If Yes, please provide a short description.

9.1.d Do patients have to pay to receive counselling on physical activity? Y/N

9.2 Is physical activity and health (health effects, determinants, effective interventions, etc.) taught in the curriculum of health professionals (e.g., nurses, medical doctors, physiotherapists, etc.)? Y/N

9.2.a Can you please comment on why you have answered No?

9.2.b If Yes, please select the type(s) of health professional(s). (check all that apply)

9.2.c Please also comment on whether it is part of undergraduate and/or postgraduate studies or both, and if it is mandatory or optional.

9.3 Does your country have a success story and/or case study related to this indicator?

9.3.a If Yes, please provide a short description and a link.
