# Supplementary material for: Physical activity promotion and health-enhancing physical activity education policy in EU healthcare: a cross-sectional survey of 27 member states
Source: BMJ Open. 2025 Aug 13;15(8):e095218. doi: 10.1136/bmjopen-2024-095218 (PMC12352183; doi:10.1136/bmjopen-2024-095218)
Supplement: Supplementary file 3 [file bmjopen-2024-095218.reviewer_comments.pdf]

PEER REVIEW HISTORY

BMJ Open publishes all reviews undertaken for accepted manuscripts. Reviewers are asked to complete a checklist review form and are provided with free text boxes to elaborate on their assessment. These free text comments are reproduced below.

ARTICLE DETAILS

Title (Provisional)

Physical activity promotion and health-enhancing physical activity education policy in EU healthcare: a cross-sectional survey of 27 member states

Authors

Leese, Callum; Whiting, Stephen; Mendes, Romeu

VERSION 1 - REVIEW

**Reviewer**	**1**
**Name**	**Mantzari, Eleni**
**Affiliation**	**King's College London**
**Date**	**13-Mar-2025**
**COI**	**None**

Very clearly written, concise manuscript. I have a few comments to hopefully increase the value of the manuscript.

Introduction

The aim of the paper is described “to analyse data…to improve understanding of the current status of policies in the European Union member states related to physical activity counselling and exercise referral schemes in health care settings and the education of health professionals in health-enhancing physical activity”. Can this be clarified? It is not clear what “the status” means. Is it purely to describe the proportion of countries that have relevant policies and include relevant education in health professionals’ curriculums If so it’s important to be clear and specific. I was under the impression that “the status” would also cover more the content of the policies and curriculums but this doesn’t seem to be the case.

Given the monitoring framework was implemented in 2013, is this the first attempt to analyse the data in such a way? Were there any similar past attempts? If so can these be described?

Methods

Methods could be elaborated more on. For example, when exactly were data collected? Do authors have any more specific information on who the survey respondents were? Was the

survey distributed to all countries at the same time? Given that data on the prevalence of policies/educations programmes of each country are based on their responses, understanding who the respondents are is especially important.

Although the list of questions is in the supplement, a brief description of these in the main text would aid the reader

Results

The results are quite descriptive in terms of the proportion of countries that include policies and include PA education in healthcare professionals' curriculums. Although this is interesting to an extent to know, having information on the content/types of the policies and curriculums would be far more beneficial. Is that possible?

With regards to education specifically, authors report the proportion of countries that report including physical activity and Health Education in Health Professional Curriculum. However, it is not entirely clear what this it refers to. Does it involve education/training on interventions and in delivery of behaviour change techniques to promote PA? Or just on how PA influences health etc? The content and scope of this education needs elaborating.

Discussion

The authors acknowledge that one of the study limitations is that results are based on responses from individual experts rather than what countries actually do. I personally find this quite concerning, least because not enough information is given about the respondents to ascertain whether they are appropriate experts. I realise it might be quite resource intensive but could some cross-validation have been conducted to assess the extent to which obtained data from the survey actually correspond to things that are implemented in countries? At least for a random sub-sample of the countries?

**Reviewer**	**2**
**Name**	**Ramirez Varela, Andrea**
**Affiliation**	**University of Texas McGovern Medical School**
**Date**	**26-Mar-2025**
**COI**	**None**

The study explores the prevalence of physical activity counselling programs in healthcare settings and the integration of health-enhancing physical activity (HEPA) into health professional curricula.

I enjoyed reading the document, which is well-written and reflects a significant amount of high-quality work. The relevance of this research is strong given the rising chronic diseases associated with sedentary lifestyles.

Please find below specific comments on this manuscript.

1. The abstract could benefit from a clearer and more explicit statement of the study's objective. In particular, the objective described in the introduction is more clearly defined, and aligning the abstract with this could provide readers with a clearer context.

2. The methods section could be improved by including the variables used in the study's survey in the main text rather than in the supplementary material.

3. The question of why policy implementation was not assessed stands out. Understanding the real-world implementation of policies is crucial to gaining a full picture of how these programs are functioning across different countries in the EU context. It would be beneficial to include a discussion on the implementation challenges of these policies to better understand the gap between theory and real-world practice.

4. The inclusion of all 27 EU Member States is a notable strength. This broad representation provides a comprehensive view of physical activity practices and challenges across Europe. Additionally, the use of the HEPA Monitoring Framework enhances the credibility of the findings, and the descriptive analysis offers a clear picture of the current state of physical activity counselling and HEPA education.

5. While the study presents a solid overview, there is potential for a deeper analysis. For instance, more specific insights into how different countries are implementing physical activity counselling and HEPA education could strengthen the findings. A detailed discussion of the successes and challenges experienced by individual countries would help provide a better understanding of what strategies are most effective and why. Regional differences or common trends across countries could also be explored to provide more details.

6. The paper identifies barriers to physical activity counselling and HEPA education but lacks specific, actionable recommendations for policy changes. Including case studies of successful programs, such as Slovenia, would provide useful examples for other countries aiming to improve physical activity integration in healthcare settings.

7. The concept of the Physical Activity Care Continuum, which connects patient screening, prescription, and referral to community resources, could be highlighted in the discussion. This model emphasizes the importance of creating a feedback loop between patients and healthcare providers, which could enhance the overall integration of physical activity into healthcare practices. Referencing successful international models could offer valuable lessons for the EU context.

8. Additionally, a discussion on the barriers to physical activity prescription, such as time constraints, reimbursement issues, and inadequate professional training, would provide insight into the underlying reasons for the challenges faced in implementation in the EU context.

9. Incorporating an implementation science approach could also improve the paper by

focusing on how to integrate physical activity counselling more efficiently into healthcare systems. A discussion of the cost-effectiveness of including physical activity in healthcare programs would further strengthen the argument for policy change.

10. The study's reliance on a single representative from each country might introduce some bias. Triangulation of the results with additional sources could increase the reliability of the findings.

11. Providing national-level recommendations and discussing potential changes to public health systems would help create a clearer roadmap for improving physical activity integration into healthcare.

This study provides valuable insights, but it could be enhanced by exploring the EU context in more detail, providing actionable policy recommendations, and expanding the results from the survey for the inclusion of case studies.

VERSION 1 - AUTHOR RESPONSE

Reviewer: 1

1. Introduction: The aim of the paper is described "to analyse data...to improve understanding of the current status of policies in the European Union member states related to physical activity counselling and exercise referral schemes in health care settings and the education of health professionals in health-enhancing physical activity". Can this be clarified? It is not clear what "the status" means. Is it purely to describe the proportion of countries that have relevant policies and include relevant education in health professionals' curriculums If so it's important to be clear and specific. I was under the impression that "the status" would also cover more the content of the policies and curriculums but this doesn't seem to be the case.

Thank you. This has been re-written, clarifying that the paper reports on the current policies in EU Member States.

"This report aims to analyse data collected through the HEPA Monitoring Framework to describe the proportion of European Union member state countries that have relevant policies related to physical activity counselling and exercise referral schemes in health care settings and the education of health professionals in health-enhancing physical activity."

2. Given the monitoring framework was implemented in 2013, is this the first attempt to analyse the data in such a way? Were there any similar past attempts? If so can these be described?

Thank you for the comments. The framework was implemented initially in 2013, but only since 2015 has the European Commission (in collaboration with the WHO Regional Office for Europe) been monitoring the implementation of HEPA policies in EU Member states(1-3). This has been done in three-yearly cycles with reports published by WHO Regional Office for Europe.

To offer further clarity and add context we have moved and edited the following text:

"Since 2015, in three yearly cycles, the European Commission in collaboration with the WHO Regional Office for Europe have been monitoring the implementation of HEPA policies in EU Member states(23-25). The published reports offer a limited overview of HEPA policies. Of relevance they report 75% (n=21) of countries had a national programme or scheme for the provision of counselling on PA by health professionals in 2021, compared to 67% (n=18) in 2018. Similarly, in 2021, PA for health was included in the curriculum of one or more types of health professional in 78% (n=21) of countries, compared to 61% (n=17) in 2015 and 79% (n=22) in 2018."

Given the previously limited data availability and reported on HEPA policies in health-care settings, this report aims to further analyse data collected through the HEPA Monitoring Framework to describe the proportion of European Union member state countries that have relevant policies related to physical activity counselling and exercise referral schemes in health care settings and the education of health professionals in health-enhancing physical activity"

"As previously described, the implementation of HEPA policies of in EU Member states has been monitored in 2015, 2018 and 2021 (23-25)."

3. Methods could be elaborated more on. For example, when exactly were data collected? Do authors have any more specific information on who the survey respondents were? Was the survey distributed to all countries at the same time? Given that data on the prevalence of policies/educations programmes of each country are based on their responses, understanding who the respondents are is especially important.

Thank you for the comment. The following text has been added to offer more clarity as highlighted above. Unfortunately, the contact details and information for all the individuals within the Focal Point Network is not publicly available.

“For the 2021 round of data collection, an electronic English language questionnaire was created by experts within the WHO Office for the European Region in January 2021 using LimeSurvey software. It was distributed to the 27 EU members states in the WHO European Region through the European Union Physical Activity Country Focal Points Network at the beginning of March 2021. This is a network of experts officially nominated by their governments to support data collection on HEPA promotion at the national level. These experts usually work in national ministries of health, ministries of sport, or related national agencies. The survey was open for two months and during this time the WHO maintained a helpdesk and held three webinars to provide guidance to officials nominated as part of the Focal Point Network. On closing all 27 countries had responded. WHO staff then manually checked all responses and contacted corresponding focal points if further clarification was needed.”

5. Although the list of questions is in the supplement, a brief description of these in the main text would aid the reader.

Thank you. The following text has been added:

“The questionnaire (detailed in Supplementary File 2) asked about the existence of national guidance or programmes for PA counselling by healthcare professionals, the involvement of various health professions, financial incentives, and whether patients incur costs for such services. Additionally, the survey covered the integration of HEPA in the curricula of health professionals, specifying professional groups, study levels, and whether such education is mandatory or optional. Respondents were also invited to share relevant success stories or case studies”

6. The results are quite descriptive in terms of the proportion of countries that include policies and include PA education in healthcare professionals’ curriculums. Although this is interesting to an extent to know, having information on the content/types of the policies and curriculums would be far more beneficial. Is that possible?

Thank you for the suggestion. Unfortunately, the data is limited to that which we have presented. However, this work could form the foundation for a further more explorative study.

7. With regards to education specifically, authors report the proportion of countries that report including physical activity and Health Education in Health Professional Curriculum. However, it is not entirely clear what this it refers to. Does it involve education/training on interventions and in delivery of behaviour change techniques to promote PA? Or just on how PA influences health etc? The content and scope of this education needs elaborating.

We agree that clarification is needed regarding what is encompassed by "physical activity and health education" within health professional curricula. As outlined in Supplementary File 2, the relevant question (9.2) asked whether *"physical activity and health (health effects, determinants, effective interventions, etc.) is taught in the curriculum of health professionals"*, with further follow-up questions on the type of health professionals involved, the level of education (undergraduate/postgraduate), and whether the content is mandatory or optional. We have added the following to the discussion to try and outline the limitations of this study:

"Firstly, all answers are based on a single expert response from national governments who may have a limited overview. The quality and depth of these responses are inherently dependent on the knowledge of the focal points within the EU Physical Activity Focal Point Network. Given the variation within educations institutions within a single country, the responses are also likely influenced by the access to detailed curricular information from relevant professional training institutions that the local focal point responder had available. Therefore, while we report the proportion of countries indicating inclusion of HEPA, we acknowledge that the scope likely ranges from general education on PA and its health impacts, to more applied or clinical content such as counselling techniques or prescription practices.

Furthermore, study data is limited to qualitative answers from surveys with no direct information available on what countries actually do. Challenges in interpretation of

answers exist, with responses ‘no’ or ‘-‘ either being an affirmative response or an indication that the responding expert is not sure if policy exists. In addition the study method may have limited response depth. For example, the survey did not provide a detailed breakdown of specific educational content areas, such as whether curricula included training on behaviour change techniques, patient counselling, or intervention delivery.”

8. Discussion: The authors acknowledge that one of the study limitations is that results are based on responses from individual experts rather than what countries actually do. I personally find this quite concerning, least because not enough information is given about the respondents to ascertain whether they are appropriate experts. I realise it might be quite resource intensive but could some cross-validation have been conducted to assess the extent to which obtained data from the survey actually correspond to things that are implemented in countries? At least for a random sub-sample of the countries?

Thank you for raising this point. We agree that the validity and representativeness of survey responses are critical considerations, particularly when interpreting national-level data.

As outlined in the discussion section of the manuscript, we have acknowledged that a key limitation of our study is that the results are based on the responses of individual experts rather than direct audits or implementation data from countries.

By using the EU Physical Activity Focal Point Network—an established mechanism coordinated by the European Commission and WHO Europe—as the primary channel for data collection we hope to minimise this risk. Each respondent was officially nominated by their national government and embedded within national institutions (typically ministries of health, sport, or public health agencies). While they are recognised as focal points for PA policy, they are not expected to have all information personally and were encouraged to consult with colleagues and relevant stakeholders to ensure accurate and comprehensive responses.

We appreciate the reviewer's suggestion regarding cross-validation. While we agree that this could further strengthen the findings, resource constraints and the scope of the current project limited our ability to systematically verify implementation across all or even a subset of countries.

We have revised the discussion, as outline above, to outline the limitations of this method and the potential for future studies to undertake targeted validation exercises. This includes the addition of the following line "A first step to address this would look to perform a more thorough evaluation of individual countries policies, allowing for trangulation with these results".

Reviewer: 2

1. The abstract could benefit from a clearer and more explicit statement of the study's objective. In particular, the objective described in the introduction is more clearly defined, and aligning the abstract with this could provide readers with a clearer context.

Thank you. This has been changed to as follows:

"Analyse data collected through the WHO Regional Office for Europe to describe the proportion of European Union member state countries that have relevant policies related to physical activity counselling and exercise referral schemes in health care settings and the education of health professionals in health-enhancing physical activity."

2. The methods section could be improved by including the variables used in the study's survey in the main text rather than in the supplementary material.

Thank you for suggesting this. As per reviewer 1's comments we have added further detail to the text (in addition to supplementary file 2).

3. The question of why policy implementation was not assessed stands out. Understanding the real-world implementation of policies is crucial to gaining a full picture of how these programs

are functioning across different countries in the EU context. It would be beneficial to include a discussion on the implementation challenges of these policies to better understand the gap between theory and real-world practice.

In the current study, our primary aim was to review the *existence* of national policies and programmes related to physical activity promotion across EU Member States. The focus was therefore on mapping the presence of guidance and policy rather than evaluating how these policies are operationalised.

We acknowledge that the presence of a policy does not guarantee its effective implementation. As pointed out, understanding implementation challenges—including resource limitations, institutional fragmentation, or lack of enforcement—is key to interpreting the practical impact of national strategies. Therefore, in response to this we have edited the discussion as follows:

"Building on previous work(26), a recent publication Whiting and colleagues (27) has highlighted the ongoing gap between PA policy and PA implementation. There are a number of challenges including a lack of evidence to inform PA policy (28), poor translation of effective research into practice (including accounting for country and cultural specific factors) and a failure to implement effective strategies(27). This maybe further complicated by a lagtime between implementation and results, and a large number of confound factors, including: economic develoipments, social trends, climate change and more recently the COVID-19 pandemic(27)."

4. The inclusion of all 27 EU Member States is a notable strength. This broad representation provides a comprehensive view of physical activity practices and challenges across Europe. Additionally, the use of the HEPA Monitoring Framework enhances the credibility of the findings, and the descriptive analysis offers a clear picture of the current state of physical activity counselling and HEPA education.

Thank you.

5. While the study presents a solid overview, there is potential for a deeper analysis. For instance, more specific insights into how different countries are implementing physical activity counselling and HEPA education could strengthen the findings. A detailed discussion of the successes and challenges experienced by individual countries would help provide a better understanding of what strategies are most effective and why. Regional differences or common trends across countries could also be explored to provide more details.

> This would be a valuable piece of work which the authors would support, and with time and capacity be keen to engage in. However, the remit of this work was narrow and the data-available from the WHO limited to what has been presented. Thank you, however, for the suggestion.

6. The paper identifies barriers to physical activity counselling and HEPA education but lacks specific, actionable recommendations for policy changes. Including case studies of successful programs, such as Slovenia, would provide useful examples for other countries aiming to improve physical activity integration in healthcare settings.

> Thank you for this suggestion. Specific case studies have been included in Supplementary File 1, including Austria, Romonia, Czechia, Germany, Bulgaria and France. This was with the hope of offering, as you highlight, actionable recommendations for policy changes. If, at the request of the editor, it is felt this is best included somewhere within the main body of the text then we are more than happy to do so.

7. The concept of the Physical Activity Care Continuum, which connects patient screening, prescription, and referral to community resources, could be highlighted in the discussion. This model emphasizes the importance of creating a feedback loop between patients and healthcare providers, which could enhance the overall integration of physical activity into healthcare practices. Referencing successful international models could offer valuable lessons for the EU context.

> Thank you for this suggestion. I have never heard of the physical activity care continuum, and on searching could find information related to the physical activity advice

continuum which illustrates a point made in the discussion. Consequently, the following text has been added, alongside a practical example from the United Kingdom:

"In discussing the physical activity advice continuum, Freene and colleagues (34) highlight the importance of physical activity promotion coming from the full spectrum of care providers regardless of discipline or availability of time. In the development of the 'Active Hospitals' toolkit(35), Moving Medicine and the Faculty of Sport and Exercise Medicine are actively trying to address this in the United Kingdom, complementing pre-existing initiatives in primary care(36, 37)."

If you feel further action in needed, and are able to direct to literature on the physical activity care continuum then we are more than happy to edit further.

8. Additionally, a discussion on the barriers to physical activity prescription, such as time constraints, reimbursement issues, and inadequate professional training, would provide insight into the underlying reasons for the challenges faced in implementation in the EU context.

Thank you for this suggestion, the following text has been added to address this:

"Despite this, PA promotion in primary care remains poorly implemented. For example 72% of GPs in England self-report that they do not deliver PA promotion(12). A recent systematic review explored factors associated with delivery and receipt of PA advice and support in primary care(15). Key practitioner barriers included a lack of time and training/guidelines, and a perceived lack of patient motivation/adherence to PA advice. Additionally, organisational factors included economic constraints, and a lack support from management. The challenges are consistent across a number of other reviews(33, 34). These findings, and particularly the organisational challenges, highlight the need for policy implementation and support."

9. Incorporating an implementation science approach could also improve the paper by focusing on how to integrate physical activity counselling more efficiently into healthcare systems. A discussion of the cost-effectiveness of including physical activity in healthcare programs would further strengthen the argument for policy change.

Thank you for this suggestion and we agree that the use of an implementation framework (for example RE-AIM) would enable the practical outcomes to add in PA

counselling integration. However, the aim of this paper was to describe the proportion of European Union member state countries that have relevant policies related to physical activity counselling and exercise referral schemes in health care settings and the education of health professionals in health-enhancing physical activity. As such, given the descriptive nature of the work, we feel the use of an implementation framework of limited value. However, we see this as a foundation/mandate for further work, including the development of implementation strategies.

Thank you for your suggestion regarding the cost effectiveness of physical activity in healthcare programmes. We have added the following sentence to address this:

"To improve the mandate for this further research could look to create a robust economic argument for the inclusion of teaching within healthcare curriculums, addressing a current evidence gap."

10. The study's reliance on a single representative from each country might introduce some bias. Triangulation of the results with additional sources could increase the reliability of the findings.

Thank you for raising this point. We agree that the validity and representativeness of survey responses are critical considerations, particularly when interpreting national-level data. We hope to have answered this question in response to a similar comment from reviewer 1.

11. Providing national-level recommendations and discussing potential changes to public health systems would help create a clearer roadmap for improving physical activity integration into healthcare.

Thank you for this suggestion. We have included, as described above, a number of case studies within supplementary file one which provide a number of national-level policies. Regarding the potential changes to public health systems, we hope through the text added in response to previous comments (specifically about the implementation gap and challenges to practitioner time) that we have created a clear mandate for action.

This study provides valuable insights, but it could be enhanced by exploring the EU context in

more detail, providing actionable policy recommendations, and expanding the results from the survey for the inclusion of case studies.

Thank you. We hope in the preceding comments to have actioned your comments where able and significantly improved the manuscript.

Thank you once again for your comments, and your consideration of this article for *BMJ Open*. We hope that the above has offered clarity and changes made improve the reading of the manuscript. Should any further clarity or changes be required please do not hesitate to contact us.

Many Thanks

Dr Callum Leese

1. WHO. Physical Activity Factsheets for the 28 European Union Member States of the WHO European Region. Copenhagen: WHO Regional Office for Europe; 2018 [Available from: https://www.who.int/europe/publications/i/item/EUR-RC71-R14.
2. WHO. 2021 Physical Activity Factsheet for the European Union Member States in the WHO European Region Copenhagen: World Health Organsiation; 2021 [
3. WHO. Factsheets on Health-Enhancing Physical Activity in the 28 European Union Member States of the WHO European Region Copenhagen: World Health Organisation; 2015 [
